# Single‐Cell RNA Sequencing for Precision Oncology: Current State-of-Art

**DOI:** 10.1007/s41745-020-00178-1

**Published:** 2020-06-02

**Authors:** Justine Jia Wen Seow, Regina Men Men Wong, Rhea Pai, Ankur Sharma

**Affiliations:** Genome Institute of Singapore, 60 Biopolis St, Singapore, 138672 Singapore

## Abstract

Tumors exhibit genetic and phenotypic diversity leading to intra-tumor heterogeneity (ITH). Further complex ecosystem (stromal and immune cells) of tumors contributes into the ITH. This ITH allows tumors to overcome various selection pressures such as anti-cancer therapies and metastasis at distant organs. Single-cell RNA-seq (scRNA-seq) has provided unprecedented insights into ITH and its implications in drug resistance and metastasis. As scRNA-seq technology grows and provides many new findings, new tools on different programming platforms are frequently generated. Here, we aim to provide a framework and guidelines for new entrants into the field of scRNA-seq. In this review, we discuss the current state-of-art of scRNA-seq analysis step-by-step including filtering, normalization and analysis. First, we discuss the brief history of experimental methods, followed by data processing and implications in precision oncology.

## Introduction

Most of the cells in the human body have the identical genetic material; despite that, at the level of gene expression, these cells show exceptional variability^38.^ For example, the cellular composition and transcriptome of liver (a metabolic organ) are very different from brain^31^. Moreover, there is heterogeneity in cellular population within the same organ i.e. liver is made up of hepatocytes, cholangiocytes and a variety of other stromal cells such as endothelial cells, fibroblasts and immune cells^28^. Therefore, transcriptional profiling by bulk sequencing methods provides the average transcriptome of different cell types. However, in the last decade, we have gained exceptional advances in technologies to profile the transcriptome of individual cells^54^. This paves the way for understanding the *transcriptional heterogeneity*

**Transcriptional heterogeneity**: Heterogeneity between mRNA content of individual cells is an inherent feature of dynamic cellular processes. The scRNA-seq provides an opportunity to understand the transcriptional heterogeneity in disease and developmental contexts

of different cell types in seemingly homogenous population.

Like any other emerging technology, there are challenges that we need to keep in mind especially when applying scRNA-seq on complex clinical samples. One of the major challenges is the dissociation and recovery of all the cell types within a tissue before proceeding with single-cell capture. During enzymatic digestion of solid heterogenous tissues, some populations like immune cells (lymphocytes) are easy to dissociate when compared to epithelial cells (hepatocytes) with tight junctions [44. On the other hand, harsher dissociation conditions may allow the recovery of majority of cells but at the expense of damaging the quality and quantity of RNA in these cells. After dissociation, the next step is sequencing, followed by data processing and filtering for good quality of cells. Different cell populations in a tissue may have different RNA and mitochondrial content. Therefore, filtering steps should be optimized for each tissue type. Finally, based on the biological question and the experimental set-up, these data can be probed to understand cellular trajectories and interactions. In this review article, we will briefly discuss challenges and best practices of single-cell RNA-seq analysis in the view of precision oncology.

## Experimental Set-up

The querying of single cells started with isolating individual cells by limiting dilution or mouth pipetting^50^. However, given the low throughput and tedious nature of experimental set-up, flow sorting soon became the method of choice. In early 2010s, invention of microfluidics-based isolation technology provided the semi-automated and moderate throughput solution^55^ and in 2015/16, *droplet-based methods *

**Figure 1:**
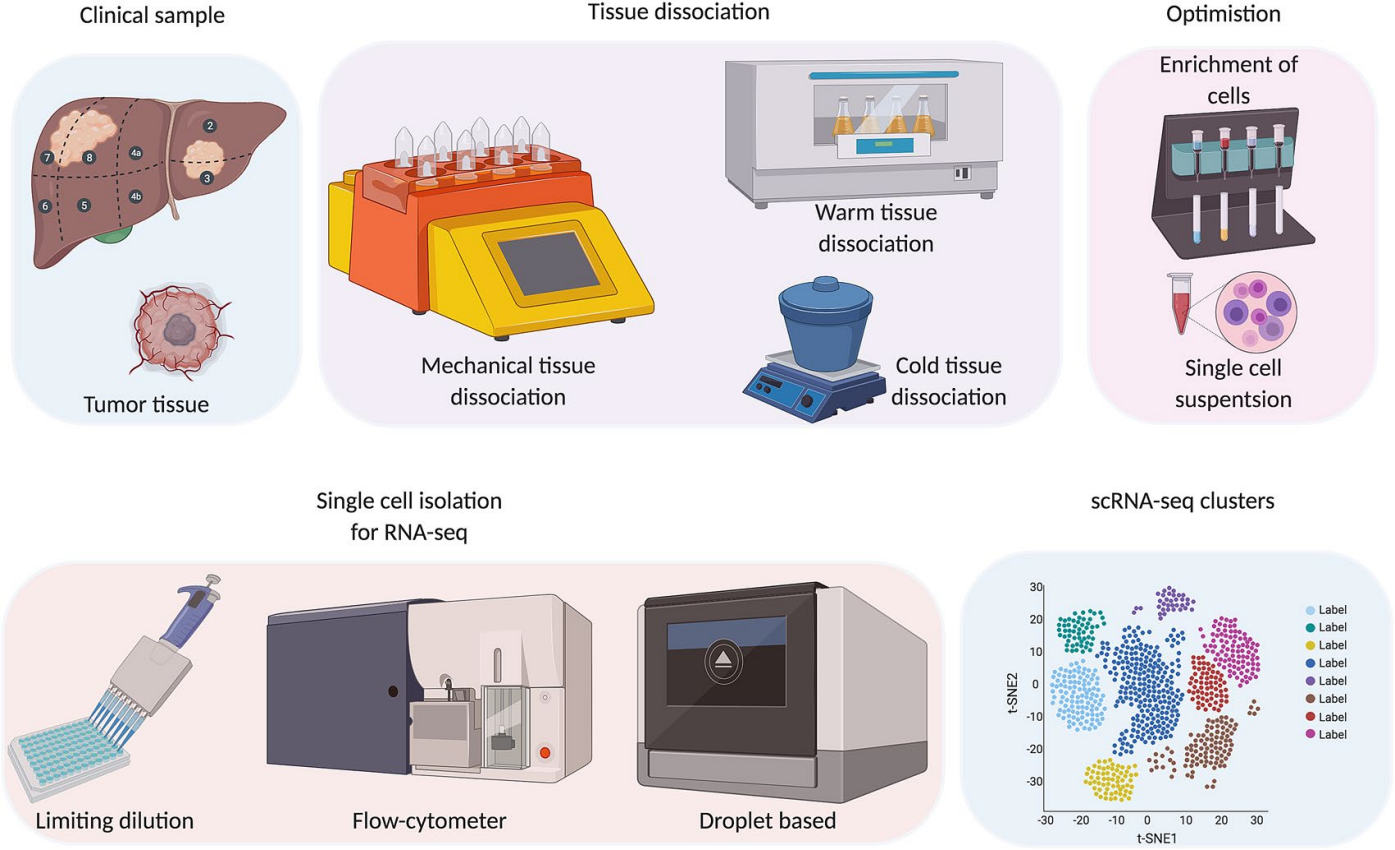
Schematic of scRNA-seq experimental set-up from collection of clinical samples, tissue dissociation, and optimization to obtain desired cell type, capturing of single cells to data acquisition.

**Droplet-based methods**: Microfluidics based methods to generate droplets for single cell isolation

(by 10 × Genomics Inc.) revolutionised the field of single-cell genomics^62^. There are a few challenges that can be faced while performing single-cell experiments in lab. First, the data obtained from single cell experiments highly depend on the type of sample and dissociation method used. PBMCs and cell lines are easy to dissociate, while complex solid tissues could be challenging due to the heterogeneity of the samples. A brief schematic of scRNA-seq experimental set-up is depicted in Fig. 1.

The condition of samples employed for single-cell experiment plays an important role. It is not always feasible to perform experiment on fresh tissue samples, especially in case of clinical samples. Such samples can be cryopreserved as single-cell suspension in appropriate freezing media (DMSO/FBS). Moreover, dissociation protocols vary from digestion by collagenase at 37 °C to cold dissociation by protease at 4 °C^10^. Additionally, the incubation time for digestion varies depending on the protocol and nature of the tissue. Some recent studies have systematically evaluated the impact of cryopreservation on cellular composition and transcriptional profiles of solid tissues^1^, ^15^, ^51^. Additionally, rare cell types can be enriched by application of flow cytometric or magnetic-sorting techniques^33^. Majority of scRNA-seq approaches provide the steady state kinetics of mRNA (messenger RNA) expression without deeper insights into transcriptional dynamics of cells. However, a recent method called scSLAM-seq (single-cell, thiol-(SH)-linked alkylation of RNA for metabolic labelling sequencing) profiles the transcriptional activity at the single-cell resolution which can help in differentiating old and new RNA for thousands of genes^14^ A very recent method *SMART-Seq3*

**SMART-Seq**: A single-cell sequencing method with switch mechanism at the 5' end of templates and improved read coverage across transcripts.

provides the allele and isoform resolution in scRNA-seq approach^17^.

## Data Preprocessing and Quality Control

scRNA-seq inherits a large number of technologies from bulk RNA-sequencing methods, including open source RNA-sequencing alignment tools such as STAR^12^, Salmon^34^, and kallisto^4^. One of the most popular and user-friendly scRNA-seq methods is the droplet-based solution from 10 × Genomics. Raw data obtained from sequencing systems are in form of bcl, fastqs, and bam files. Currently, the most favored method for alignment of reads is Cell Ranger, which is based on the STAR pipeline. However, Cell Ranger is more computationally intensive. Recently, it has been proposed that for a large reference genome such as the human reference genome, kallisto|bustools may reduce the time and computational power required for alignment^32^. Cell Ranger uses counts while kallisto|bustools and Salmon use the *pseudoalignment*

**Pseudoalignment**: this approach determines each read’s compatibility with transcripts in sequencing data.

technique which may provide an advantage for large datasets.

Analyzing scRNA-seq data is challenging due to its multidisciplinary facet of data preprocessing. Therefore, a long list of statistical methods has been built and tested on different datasets generated. Current state-of-art and popular scRNA-seq toolkits are Scanpy^58^ and Seurat^6^. There is not one standardized quality control pipeline for data clean up. Generally, data cleaning retains viable good quality cells by filtering out low-quality cells through measuring variables such as the number of UMI counts per cell, UMI counts per genes, and the proportion of mitochondrial genes expressed. Common practices for single-cell data analysis includes removing empty droplets and cells that have low count and a high proportion of mitochondrial genes. Generally, cells that are expressing less than 100–300 count/cell, 10–30 count/gene and more than 20% mitochondrial genes are excluded^42^. These can be easily visualized through violin plots to determine appropriate cutoffs. It is important to exclude these cells as a dying cell might release cytoplasmic RNA in reaction mixture and cause *ambient RNA contamination*

**Ambient RNA contamination:** Background contamination of RNA from dying single cells in droplet-based methods.

For example, in liver cancer studies, ALB, HBA, HBB, and MALAT1 are known to be some of the contaminant genes found ubiquitously in the surrounding. Computational approaches tools such as SoupX^61^ and Souporcell^19^, ^20^ can help in detecting ambient RNA and removal of unwanted cells. Conversely, cells that are expressing too many genes might represent doublets that are captured in data processing. However, there are more sophisticated algorithms implemented such as Scrublet^60^, DoubletFinder^30^, and DoubletDecon^11^ to remove outliers and doublets.

All variables should be considered jointly while QC steps are taken. Unfortunately, there are no best general data cleaning thresholds that can be set as each data has its own properties to focus on. Usually the best data cleaning reflects on the annotation of cell types. Note that these cutoffs are the same throughout the dataset; however, different cell types express different number of genes. For example, immune cells yield lower number of genes as compared to other cell types and cancer cells usually generate more genes as compared to non-cancerous cells (Seow and Sharma unpublished data). Besides that, different technologies capture different number of cells, and 10X datasets usually contain a higher number of dropouts, whereas Smart-seq2 captures more genes/cell. Therefore, a common practice is to start off with default cutoffs, working through the downstream analyses, annotating the clusters, and ending off with revisiting and reassessing QC cutoffs accordingly.

## Normalization

The capture of mRNA from individual cells varies within and across the samples; therefore, normalization of data helps in overcoming this bias. Methods such as Scanpy^58^, Seurat^6^, and CellRanger employ the same global library normalization method. This method first multiplies each cell by a scale factor of 10e^4^ and a *natural log transform*

**Natural log transform**: To transform skewed data to approximately conform to normality. This results in a data set that is roughly symmetric and often roughly normal

each value. This helps in handling data that biases towards large values and does not diminish small values. Some methods scale data to unit variance, mean value, and standard deviation of a maximum of 10 preventing over domination of certain genes. The practice of regressing out biological covariates such as cell cycle effects, mitochondrial genes, and count depth is still in debate of whether they are helpful or not as these factors may represent biological processes. Since not all biological processes are understood, regressing one or two biological technicalities might enhance or mask the others.

Subsequent to normalization, a common problem with scRNA-seq datasets is batch effects. This is where a variation between scRNA-seq datasets can be visualized based on samples prepared in separate batches. This can be common in cancer samples as different patients, tissue types, and treatment conditions can lead to batch effect. Some algorithms such as ComBat have been developed to correct for these effects^7^. However, the most popular algorithms for scRNA-seq *batch correction*

**Batch correction**: scRNA-seq datasets generated across different conditions or from technologies that contain batch specific systematic bias leading to batch-effect. Removal and correction of this effect by data integration is batch correction.

are Harmony^24^, LIGER^57^, and Seurat 3^47^, as it has been shown to outperform existing batch correction methods in most datasets^53^. Currently, Cell Ranger 3.0 by 10X Genomics has also implemented the *mutual nearest neighbours*

**Mutual Nearest Neighbours**: a pair of cells from each batch is contained in each other's set of nearest neighbours

(MNN) algorithm^18^ to correct for its different chemistries. In some cases where the batches are more widely different from each other such as ones with different tissue types, or different chemistries, MNN batch correction may not be sufficient. Thus, integration methods such as Seurat^47^, LIGER^57^, Harmony^24^ and BBKNN (Polanski et al., 2020) can be used to correct for batch effects allowing for better integration of scRNA-seq datasets.

## Clustering and Annotation

A large-scale scRNA-seq atlas can contain around a million cells^16^. To condense our analyses and determine the identities of cellular landscape, clustering is employed to partition single cells into groups based on similarities in gene expression pattern. There are a variety of clustering methods that exists; however, one of the most popular method is the k-means algorithm^23^, ^27^. First, a number of k clusters are identified, then each cell is subsequently assigned to the closest cluster^23^. However, as scRNA-seq datasets have increased in size over the number of years, community-detection-based algorithms are now being popularized for scRNA-seq clustering, specifically *K-nearest neighbours*

**K-nearest neighbours**: The k closest cells from data set used for classification and regression for single-cell RNA-seq data

graphs (KNN). This graph-based algorithm only searches for cell pairs within its neighborhood (nearest neighbors) to determine a cell’s identity, thus reducing computational time and power^27^. Currently, Louvain is one of the most popularized community detection algorithms that is implemented in Seurat and Scanpy. However, a recent comparison of Louvain and Leiden revealed that Louvain may lead to poorly connected communities and Leiden outperforms Louvain in computational speed^52^.

**Figure 2:**
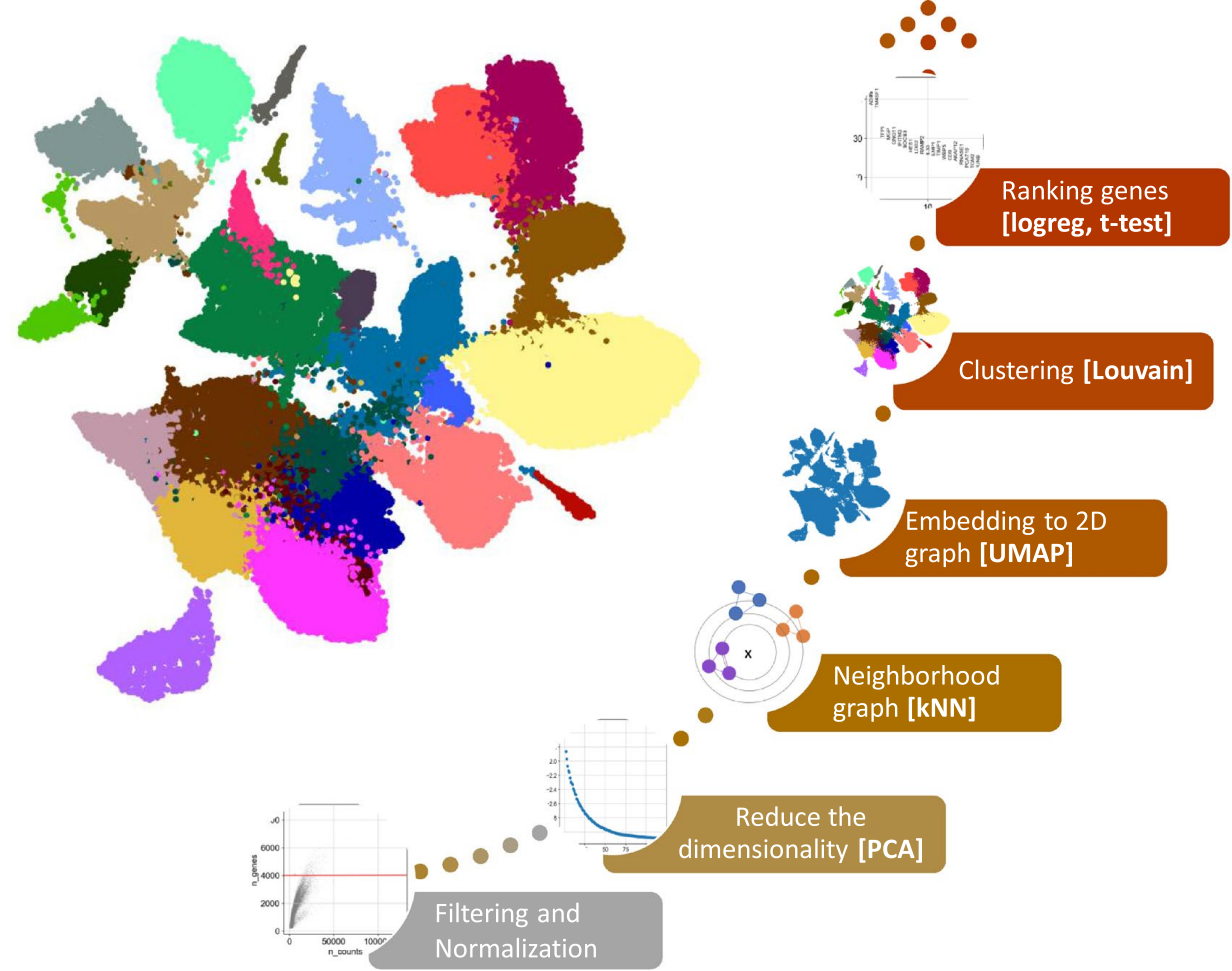
scRNA-seq from data normalization, dimensionality reduction to clustering and annotation of cell types.

Following clustering, annotation is needed to determine the cellular identity of each cluster. Similar to quality control, there are many approaches to this and not one standardized pipeline. Traditionally, identifying cell identities is done manually where a known list of differentially expressed genes for specific cell types is required. Known marker genes are plotted onto a UMAP (uniform manifold approximation and projection) and a heatmap with the differentially expressed genes which can be employed to annotate specific cell types. Another approach is to use algorithms like reference component analysis^25^ where it first broadly identifies cell identities and subsequently one can manually identify specific cell identities through differentially expressed genes. More recently, automatic annotation algorithms have begun to emerge where it can simplify or speed up this process. Seurat was identified as one of the top-performing automatic annotation algorithms in a benchmarking analysis^47^. However, a caveat of this method is that it currently can only transfer cell type labels from the reference dataset onto one other query dataset. Garnett is a different automatic annotation algorithm that uses machine learning combined with a marker list input^35^. It trains on one dataset or a subset of a dataset and can transfer cell identity labels onto another and its performance depends heavily on the marker list which leads to better annotations. A brief workflow of scRNA-seq normalisation and clustering is depicted in Fig. 2.

## Trajectory Inference

**Figure 3:**
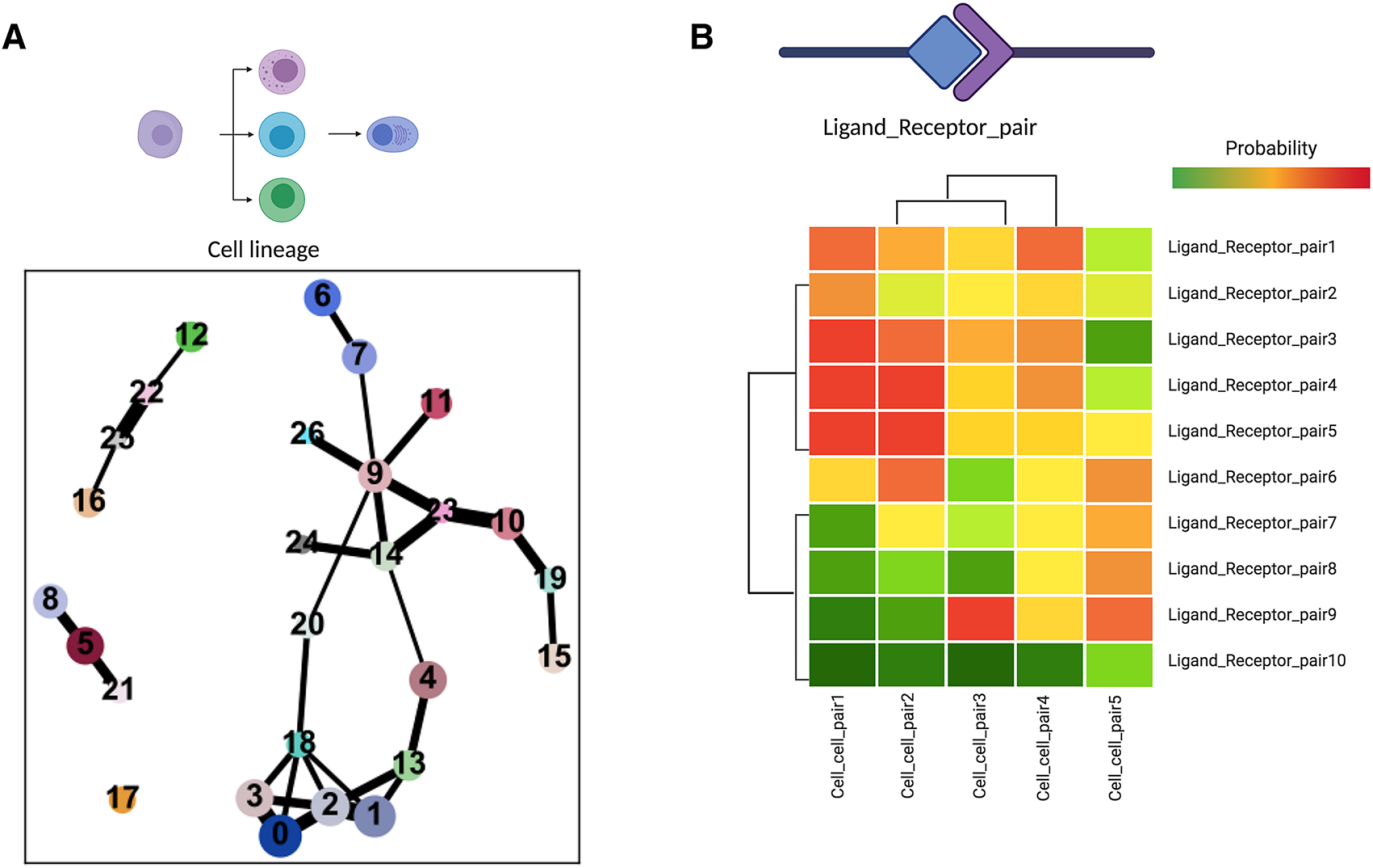
Implication of scRNA-seq data in (**a**) inferring cellular trajectories and (**b**) ligand–receptor interactions.

Cells are lysed during scRNA-seq preparation; therefore, we can only capture static timepoints from biological processes. To model the dynamics, trajectory inference can be employed to transform discrete models such as clusters into a continuous one (Fig. 3a). Particularly in cancer, trajectories of cancer cell lines can be used to identify whether there is a continuity or discontinuity in cell states^45^. The trajectory inference can indicate the mode of tumor evolution i.e. clonal selection or adaptation (cellular reprogramming)^43^^–^45. In early methods of trajectory inference, algorithms prioritized ordering cells correctly over determining best-fit trajectory models^41^. However, for more complex biological processes such as cancer plasticity, these earlier methods based on fixed topology or maximum parsimony are not optimal for modeling cellular trajectories. Since the development and popularization of Monocle^55^, term “*pseudotime*”

**Pseudotime**: Extraction of latent temporal features from single-cell RNA-seq data sets to comprehend dynamic biological processes such as cell fate transition from time A to B.

has gained the momentum and subsequently a number of trajectory inference algorithms have been developed^27^. A recent benchmarking analysis compared 45 existing trajectory inference algorithms on multiple datasets (https://dynaverse.org/)^41^. This determined slingshot^46^ to be an outstanding candidate for simple trajectories, while PAGA (partition-based graph abstraction)^59^ was the best algorithm for complex trajectories. PAGA preserves existing clustering information to minimize transcriptional changes between neighboring cell types when inferring trajectory and utilizes clusters as nodes and the computed connections between clusters as edges^59^. However, a caveat of these trajectory inference algorithms is the trajectories generated that do not have to mimic biological processes and, therefore, additional evidence should be collected to validate biological insight from trajectory inferences.

A prime example of additional information that can be embedded with trajectory inferences is splicing information^29^. RNA velocity calculates the change in the state of a cell over time by extracting unspliced and spliced mRNA reads from scRNA-seq data. This is used to infer future directionalities of single cells. Thus, algorithms like Velocyto^29^ and scVelo^3^ are currently being popularized in the field as it can calculate velocities and project them onto UMAPs with existing clustering information. This information can be represented by velocity grid plots where longer arrows correspond to large changes in gene expression and shorter arrows represent a terminally differentiated state of cell^49^. Alternatively, velocity stream plots from scVelo can minimize this information and extrapolate directionality of cell fates. This information has also been extended to gene level where candidate genes that drive differentiation can be depicted through gene-resolved velocities. Furthermore, RNA velocity can be used to calculate transition probabilities into specific subpopulations of cell types^39^. Although RNA velocity seems promising in providing additional evidence on top of inferred trajectories, a large limitation is that it currently only predicts a cell’s fate in the forward direction. With the recent development of dynamo^14^, ^37^, it can solve some of these problems as it can predict a cell’s forward and backward states with the use of scSLAM-seq^14^. Thus, as multi-omic technologies improve for single-cell analyses, traditional lineage inference may transform into building vector fields of single cells.

## Cell–Cell Interactions

Tumor ecosystem plays an important role in the process of tumorigenesis and metastasis. In early methods of analyzing cell–cell interactions, published datasets of ligand and receptor networks were used^9^, ^40^. This was coupled with gene list and bioinformatic resource such as the David GO annotation tool^9^, ^21^. In addition, to infer function of gene sets, databases such as the Gene Ontology^2^ or KEGG^22^ are employed to assign biological processes and pathways. More recently, CellPhoneDB: a repository of receptors, ligands, and their respective interactions, was developed^56^. By utilizing public databases to annotate receptors and ligands, CellPhoneDB can perform an unbiased cell–cell interaction analysis. From single-cell transcriptomic data, CellPhoneDB calculates significant receptor–ligand pairs from cluster information and differentially expressed genes. Typically, ligands and receptors expressed in more than 10% of cells in a subpopulation are considered. As a standard to determine the receptor/ligand expression levels, the algorithm iterates through the clusters of all the cells for 1000 permutations. One can use CellPhoneDB to elucidate which tumor subpopulations are expressing ligands to a corresponding receptor in a neighboring immune or stromal cell population. This information is usually represented as dot-plot or heatmap of ligand–receptor interactions (Fig. 3b). With complexity of tumor microenvironment, there is expected increase in the number of interactions in tumors compared to normal tissue^48^. The number of interactions between clusters along with their magnitudes has also been represented through a heat map or a circle plot^39^, ^56^. In the latest release of CellphoneDB v2.0^13^, it includes a comprehensive protocol and accepts a larger range of input data, making it more accessible and user friendly. One limitation of CellphoneDB v1.0 is that it only accepts gene ensemble identifiers (Ensembl IDs). One gene name could correspond to more than one Ensembl ID, thus this can be problematic when converting from gene names to Ensemble IDs. However, as the input parameters in CellphoneDB v2.0 are more flexible, it allows the user to specify their choice of gene name identifiers including gene names, Ensembl ID, and hgnc_symbol annotations.

Furthermore, another limitation of CellphoneDB is that it is currently only available for human data and not mouse data. Thus, users with mouse data have to convert mouse gene names to human gene names to use the repository. However, with the recent development of NicheNet^5^, this issue is solved as it accepts both human and mouse gene expression data. NicheNet is a method that integrates single-cell gene expression data with gene regulatory networks to predict cell–cell ligand–target interactions^5^. It ranks ligand–target interactions based on the activity of sending cells’ ligand activity on receiving cells’ gene expression^5^. This extra layer of information and ranking of significant cell–cell interactions may be beneficial for users who do not want to sort through a large list of interactions outputted by CellPhoneDB.

## Implications in Precision Oncology

**Figure 4:**
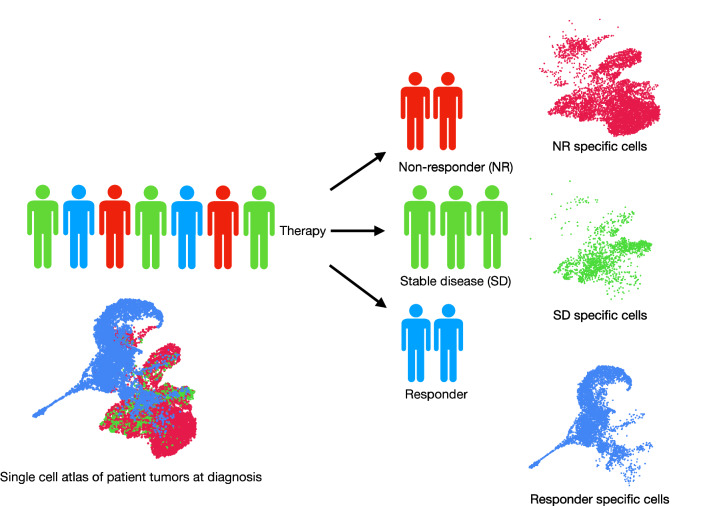
Implication of scRNA-seq in identifying disease-associated cells for precision oncology. Note the enrichment of different cellular population in responders vs non-responsers.

Recently, scRNA-seq has been applied to understand the impact of chemotherapy^45^ and immunotherapy (Sade-Feldman et al. 2018) on tumor evolution. Using scRNA-seq, we demonstrated that chemotherapy leads to either selection of pre-existing cancer-stem-like cells or adaptation into mesenchymal cell state^45^. We demonstrated that pre-existing epigenetic state guides the drug-induced epithelial to mesenchymal transition. In future, we anticipate clinical implication of scRNA-seq as a discovery tool in clinical trials. This will allow us to understand if pre-existing cell type/state can predict the response to anti-cancer therapy (Fig. 4). These discoveries will pave the way for scRNA-seq-based diagnostic tools which will facilitate the next generation of precision oncology by providing the right drug for the right patient.

## Conclusions

In last 10 years, single-cell genomics has moved from profiling gene expression in few cells to identifying novel cell populations, developmental trajectories, and cell–cell interactions. In the next decade, we anticipate development in multimodal technologies where we will be able to profile DNA, epigenome, RNA, protein, metabolome and spatial information from the same cell. This will provide unprecedented multilayered insights into functioning of a cell. We also foresee direct application of single-cell genomics in trials and decision-making process in the clinic. Currently, we are living through an unprecedented global pandemic COVID-19^26^. scRNA-seq has provided a tool to identify the cell types susceptible to viral infections^42^. Moreover, scRNA-seq has been employed to understand the immune response of individual against SARS-CoV-2^8^. Overall, scRNA-seq technology has revolutionized our understanding of the basic unit of life at single ‘cell’ resolution.
